# Clinicopathological characteristics and treatment strategies in early gastric cancer: a retrospective cohort study

**DOI:** 10.1186/1756-9966-30-117

**Published:** 2011-12-29

**Authors:** Hiroaki Ito, Haruhiro Inoue, Haruo Ikeda, Manabu Onimaru, Akira Yoshida, Toshihisa Hosoya, Kosuke Sudo, Nikolas Eleftheriadis, Roberta Maselli, Chiyo Maeda, Yoko Wada, Norimasa Sando, Shigeharu Hamatani, Shin-ei Kudo

**Affiliations:** 1 Digestive Disease Center, Showa University Northern Yokohama Hospital, 35-1 Chigasakichuo, Tsuzuki-ku, Yokohama 224-8503, Japan; 2 Department of Pathology, Showa University Northern Yokohama Hospital, 35-1 Chigasakichuo, Tsuzuki-ku, Yokohama 224-8503, Japan

**Keywords:** early gastric cancer, lymph node metastasis, endoscopic submucosal dissection

## Abstract

**Background:**

Both endoscopic and surgical approaches are employed in the treatment of early gastric cancer (EGC). The aim of this study was to establish appropriate treatment strategies for early gastric cancer.

**Methods:**

We retrospectively examined clinicopathological data of EGC patients who had undergone surgery.

**Results:**

A total of 327 patients (204 males and 123 females, mean age 63.2 years) were eligible for inclusion in the study. The median follow-up period was 31 months. Of 161 mucosal (pT1a) tumors, 87 were mainly undifferentiated and 110 had an undifferentiated component. Four patients with pT1a tumors had lymph node metastases; all these tumors were signet-ring cell carcinomas and were macroscopic type 0-IIc with ulceration, and only one of them had lymphatic invasion. Among patients with submucosal tumors, four of 43 patients with pT1b1 tumors and 37 of 123 patients with pT1b2 tumors had nodal metastases. Lymph node metastases were significantly higher in mixed undifferentiated type group than differentiated type group for both groups, pT1a-pT1b1 (p = 0.0251) and pT1b2 (p = 0.0430) subgroups. Only four of 45 patients with nodal metastases were diagnosed preoperatively by computed tomography (sensitivity 8.9%, specificity 96.2%). Nine patients with pT1b tumors had recurrence after surgery, and died. The sites of initial recurrence were liver, bone, peritoneum, distant nodes, and the surgical anastomosis.

**Conclusions:**

The incidence of nodal metastases was approximately 5% in undifferentiated type mucosal (pT1a) tumors, and higher in submucosal (pT1b) tumors. The sensitivity of preoperative diagnosis of nodal metastases in EGC using computed tomography was relatively low in this study. Therefore at present surgery with adequate lymphadenectomy should be performed as curative treatment for undifferentiated type EGC.

## Background

Gastric cancer is the fourth leading cause of cancer-related deaths worldwide [1]. Although advanced gastric cancer is often difficult to cure, early gastric cancer (EGC), which is generally recognized as a tumor with invasion confined to the mucosa or submucosa, is curable because of the low incidence of lymph node metastases [2]. The seventh edition of the International Union Against Cancer TNM guidelines defines mucosal cancers as pT1a and submucosal cancers as pT1b [3]. The third English edition of the Japanese Classification of gastric carcinoma [4] submucosal tumors are further categorizes as submucosal tumors as pT1b1 (submucosal invasion < 0.5 mm) or pT1b2 (submucosal invasion ≥ 0.5 mm). Nodal metastases are rare in pT1a tumors [5,6], but occur in 2-9.8% of pT1b1 and 12-24.3% of pT1b2 tumors [7,8]. Surgery provides excellent cure rates for EGC [9], especially limited gastrectomy with [10-12] or without [13,14] lymphadenectomy. Endoscopic treatment is a less invasive [15] alternative which is also used for the curative treatment of EGC [16], including endoscopic mucosal resection [17-20] and endoscopic submucosal dissection [15,21]. However, unsuitable use of endoscopic treatment for gastric cancer may result in local recurrence [22] and distant metastases [23] in cases which might otherwise have been curable, and should only be performed when there is an accurate diagnosis and prognosis.

The aim of this study was to investigate the optimal treatment strategy for EGC by evaluation of the clinicopathological characteristics. We focused particularly on histological type, because histological type is the only pathological factor which can be definitively diagnosed preoperatively.

## Methods

### Patients

All cases of solitary gastric adenocarcinoma which underwent curative surgery at the Digestive Disease Center, Showa University Northern Yokohama Hospital between April, 2001 and November, 2010 were retrospectively studied. The criteria for inclusion in the study were: (1) adenocarcinoma of the stomach histologically proven by endoscopic biopsy; (2) histologically solitary tumor; (3) no prior endoscopic resection, surgery, chemotherapy, or radiation therapy; (4) tumor invasion of the lamina propria or submucosa. Cases with synchronous or metachronous malignancy were excluded.

We examined relationships among histological type, tumor depth, nodal metastases, and prognosis. We also recorded the regional lymph node classification of the preoperative diagnosis. We generally performed preoperative screening for nodal metastases by computed tomography, followed by ultrasonography in cases with suspected nodal disease. Lymph nodes ≥ 1 cm in diameter on imaging were defined as metastatic nodes. We divided patients into four groups according to their pathological tumor types: (1) *differentiated type *including tumors mainly composed of well differentiated adenocarcinoma (tub1), moderately differentiated adenocarcinoma (tub2), or papillary adenocarcinoma (pap), and without poorly differentiated adenocarcinoma (por), signet-ring cell carcinoma (sig), or mucinous adenocarcinoma (muc) components; (2) *mixed differentiated type *including tumors mainly composed of tub1, tub2, or pap, and with por, sig, or muc components; (3) *mixed undifferentiated type *including tumors mainly composed of por, sig, or muc, and with tub1, tub2, or pap components; (4) *undifferentiated type *including tumors mainly composed of por, sig, or muc, and without tub1, tub2, or pap components. Disease was staged using the seventh edition of the International Union Against Cancer TNM guidelines [3].

All patient data were approved for use by the institutional review board of Showa University Northern Yokohama Hospital. Research reported in this paper was in compliance with the Helsinki Declaration.

### Statistical analysis

Fisher's exact test was used to study relationships between nodal metastases and clinicopathological findings, and logistic regression analysis was applied to determine correlations between histological groups and nodal metastases. P-values less than 0.05 were considered to indicate statistical significance. Statistical analysis was performed using JMP Statistical Discovery 9.0.2 software (SAS Institute, Cary, USA).

## Results

A total of 327 patients were eligible for inclusion in the study, including 204 males and 123 females, with a mean age of 63.2 years (range 31-89 years). The median follow-up period was 31 months.

The clinicopathological characteristics of patients are shown in Table 1.

**Table 1:** Clinicopathological findings of patients with early gastric cancer (n = 327)

Variables	Number of subjects (%)
Sex	
Male	204 (62.4)
Female	123 (37.6)
Gastrectomy	
Distal	211 (64.5)
Proximal	34 (10.4)
Total	81 (24.8)
Partial	1 (0.3)
Surgical approarch	
Laparoscopy	236 (72.2)
Hand-assist	27 (8.3)
Open laparotomy	64 (19.6)
Tumor depth *	
pT1a (m)	161 (49.2)
pT1b1 (sm1)	43 (13.1)
pT1b2 (sm2)	123 (37.6)
Lymph node metastasis ^†^	
pN0	282 (86.2)
pN1	34 (10.4)
pN2	6 (1.8)
pN3	5 (1.5)
Distant metastasis ^†^	
M0	327 (100.0)
M1	0 (0)
Main histologic type	
Differentiated	169 (51.7)
Undifferentiated	158 (48.3)
Lymphatic invasion ^†^	
L0	246 (75.2)
L1-2	81 (24.8)
Venous invasion ^†^	
V0	279 (85.3)
V1-3	48 (14.7)
Stage ^†^	
IA	282 (86.2)
IB	34 (10.4)
II	6 (1.8)
IIIA	5 (1.5)

* According to the third English edition of the Japanese Classification of Gastric Carcinoma [4].

^† ^According to the seventh edition of the International Union Against Cancer TNM guidelines [3].

Relationships between clinicopathological characteristics and nodal metastases are shown in Table 2. The only characteristic significantly associated with nodal metastases was lymphatic invasion in pT1b2 tumors.

**Table 2:** Results of univariate analyses showing relationships between clinicopathological characteristics and lymph node metastases

Variables	pT1a tumor (n = 161)		pT1b1 tumor (n = 43)		pT1b2 tumor (n = 123)	
	pN(+)	p-value	pN(+)	p-value	pN(+)	p-value
**Total**	**4/161 (2.5%)**		**4/43 (9.3%)**		**37/123 (30.1%)**	

Sex		0.6269		0.2802		0.8309
Male	3/88 (3.4%)		4/28 (14.3%)		26/88 (29.6%)	
Female	1/73 (1.4%)		0/15		11/35 (31.4%)	
Age		0.6332		0.3449		0.8432
< 65	3/91 (3.3%)		3/21 (14.3%)		16/51 (31.4%)	
65 ≤	1/70 (1.4%)		1/22 (4.6%)		21/72 (29.2%)	
Main tumor site		0.1903		0.2707		0.1129
Upper	0/19		0/3		3/21 (14.3%)	
Middle	4/89 (4.5%)		4/27 (14.8%)		17/59 (28.8%)	
Lower	0/53		0/13		17/43 (39.5%)	
Clinical macro type		0.5655		0.5579		0.4764
Depressed or excavated	3/131 (2.3%)		4/33 (12.1%)		27/96 (28.1%)	
Flat or elevated	1/30 (3.3%)		0/10		10/27 (37.0%)	
Pathological macro type		1.0000		1.0000		0.4764
Depressed	4/139 (2.9%)		4/37 (10.8%)		27/96 (28.1%)	
Flat or elevated	0/22		0/6		10/27 (37.0%)	
Ulceration		0.1287		0.3235		0.4200
No	0/72		1/23 (4.4%)		21/77 (27.3%)	
Yes	4/89 (4.5%)		3/20 (15.0%)		16/46 (34.8%)	
Main histologic type		0.1252		0.4672		0.8441
Differentiated	0/74		2/29 (6.9%)		19/66 (28.8%)	
Undifferentiated	4/87 (4.6%)		2/14 (14.3%)		18/57 (31.6%)	
Pathological tumor size		1.0000		1.0000		0.0589
≤20 mm	1/60 (1.7%)		0/7		4/28 (14.3%)	
20 mm<	3/101 (2.5%)		4/36 (11.1%)		33/95 (34.7%)	
Pathological tumor size		0.3083		1.0000		0.1730
≤30 mm	1/96 (1.0%)		2/21 (9.5%)		13/55 (23.6%)	
30 mm<	3/65 (4.6%)		2/22 (9.1%)		24/68 (35.3%)	
Lymphatic invasion ^†^		0.0731		0.5227		< 0.0001**
L0	3/158 (1.9%)		3/36 (8.3%)		4/52 (7.7%)	
L1-2	1/3 (33.3%)		1/7 (14.3%)		33/71 (46.5%)	
Venous invasion ^†^		1.0000		1.0000		0.4200
V0	4/160 (2.5%)		4/42 (9.5%)		21/77 (27.3%)	
V1-3	0/1		0/1		16/46 (34.8%)	

** *p *< 0.01.

^† ^According to the seventh edition of the International Union Against Cancer TNM guidelines [3].

We combined pT1a (m) and pT1b1 (sm1) tumors into one group because the incidence of nodal metastases was under 10% in both, and compared relationships between histological types and nodal metastases in the pT1a-pT1b1 (m-sm1) and pT1b2 (sm2) groups (Table 3). A total of 45 out of 327 patients had nodal metastases, including 8 of the 204 patients in the pT1a-pT1b1 (m-sm1) group. Rates of nodal metastases were significantly higher in the mixed undifferentiated type group than the differentiated type group (p = 0.0251).

**Table 3:** Relationships among tumor depth, histological type, and lymph node metastases

Tumor depth	Histologic type	pN(+)	Hazard ratio	95% confidence interval	p-value
m-sm1 (n = 204)	Differentiated	1/72 (1.4%)	1.000		
	Mixed differentiated	1/31 (3.2%)	2.367	0.092-61.123	0.5527
	Mixed undifferentiated	3/22 (13.6%)	11.211	1.351-233.786	0.0251*
	Undifferentiated	3/79 (3.8%)	2.803	0.350-57.357	0.3449

sm2 (n = 123)	Differentiated	11/41 (26.8%)	1.000		
	Mixed differentiated	8/25 (32.0%)	1.283	0.423-3.808	0.6539
	Mixed undifferentiated	8/14 (57.1%)	3.636	1.042-13.478	0.0430*
	Undifferentiated	10/43 (23.3%)	0.826	0.303-2.230	0.7054

* p < 0.05

Of 123 patients with pT1b2 tumors (sm2 group), 37 had nodal metastases. There was a significant association between depth of tumor invasion and nodal metastases in pT1b tumors. The incidence of nodal metastases was higher in the mixed undifferentiated type group than in the differentiated type group (p = 0.0430).

**Figure 1:**
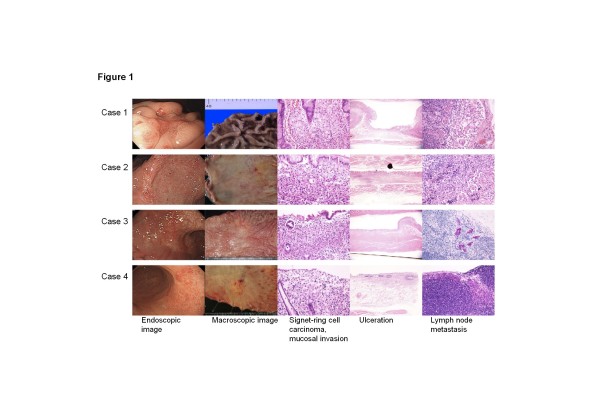
**Endoscopic, macroscopic and pathological images of mucosal tumors with lymph node metastases**. Four of 161 patients with mucosal tumors had nodal metastases. All of these patients had signet-ring cell carcinomas with ulceration. The smallest tumor was 10 mm in diameter (Case 1). One patient had non-perigastric nodal metastases along the common hepatic artery (Case 2).

The pathological characteristics of patients in the pT1a-pT1b1 (m-sm1) group with nodal metastases are shown in Table 4. All four node-positive patients with pT1a tumors had ulceration (Figure 1). The smallest tumor size was 10 mm in diameter. One patient had non-perigastric nodal metastases along the common hepatic artery.

**Table 4:** Pathological characteristics of pT1a and pT1b1 tumors with lymph node metastases

Case	Tumor depth *	Macro type	Ulceration	Tumor size, mm	Histologic type	L^†^	V^†^	Number of positive node	Follow-up time, months	Status
1	m	0-IIc	Yes	10	sig, tub2	0	0	1	97	Alive
2	m	0-IIc	Yes	42	sig, tub2, muc	0	0	1	7	Alive
3	m	0-IIc	Yes	60	sig	0	0	1	82	Alive
4	m	0-IIc	Yes	100	sig, por, tub1	1	0	1	25	Alive

5	sm1	0-IIc	No	25	tub1	0	0	1	76	Alive
6	sm1	0-IIc	Yes	25	tub2, por	2	0	4	37	Alive
7	sm1	0-IIc	Yes	31	sig	1	1	11	58	Deceased (bone metastasis)
8	sm1	0-IIc	Yes	32	por, sig	1	0	1	20	Alive

* According to the third English edition of the Japanese Classification of Gastric Carcinoma [4].

^† ^According to the seventh edition of the International Union Against Cancer TNM guidelines [3].

muc = mucinous adenocarcinoma; por = poorly differentiated adenocarcinoma; sig = signet-ring cell carcinoma; tub1 = well differentiated adenocarcinoma; tub2 = moderately differentiated adenocarcinoma.

Only 4 of 45 patients with nodal metastases were diagnosed preoperatively (sensitivity 8.9%, specificity 96.1%).

Nine patients had recurrence of cancer, and died. The initial site of recurrence was liver in three patients, bone in two, peritoneum in two, distant lymph nodes in one, and the surgical anastomosis in one (Table 5).

**Table 5:** Characteristics of cases with tumor recurrence (n = 9/327)

Case	Extent of gastrectomy	Tumor depth *	Ulceration	Main histologic type	L ^†^	V ^†^	pN ^†^	Initial recurrence site	DFS, months	OS, months	Status
1	Distal	sm1	Yes	sig	1	0	3	Bone	53	58	Deceased
2	Distal	sm2	Yes	por	1	1	1	Liver	2	3	Deceased
3	Total	sm2	Yes	por	1	0	0	Peritoneum	7	8	Deceased
4	Total	sm2	Yes	por	1	1	1	Liver	12	20	Deceased
5	Distal	sm2	Yes	tub2	1	1	1	Lymph node	12	44	Deceased
6	Distal	sm2	Yes	por	1	0	1	Liver	14	29	Deceased
7	Distal	sm2	No	por	1	0	3	Bone	19	21	Deceased
8	Distal	sm2	No	por	1	1	0	Anastomosis	23	65	Deceased
9	Total	sm2	No	tub2	1	0	0	Peritoneum	41	44	Deceased

* According to the third English edition of Japanese Classification of Gastric Carcinoma [4].

^† ^According to the seventh edition of TNM classification of the International Union Against Cancer [3].

por = poorly differentiated adenocarcinoma; sig = signet-ring cell carcinoma; tub2 = moderately differentiated adenocarcinoma; DFS = disease-free survival; OS = overall survival.

## Discussion

The most important factor to consider when selecting treatment modalities for EGC is the presence of lymph node metastases. Although nodal metastases are rare in pT1a tumors, they have been reported to occur in 2-9.8% [7,8] of pT1b1 tumors and 12-24.3% [7,8] of pT1b2 tumors. Surgical treatment is generally undertaken for pT1b2 tumors. Detailed surveys have clarified the pathological characteristics of EGC with or without nodal metastases. Nodal metastases are uncommon in differentiated type mucosal tumors [5,6,24] and in undifferentiated type mucosal tumors smaller than 20 mm in diameter without lymphatic invasion, venous invasion, or ulceration [5,6,24].

Some limitations of this study should be considered. As the patients in this study were excluded from endoscopic treatment due to the possibility of nodal metastases, the incidence of nodal disease might be higher in this group than the overall incidence in a group which includes the patients who underwent endoscopic treatment. In this study, the incidence of nodal metastases was 2.5% in pT1a, 9.3% in pT1b1, and 30.1% in pT1b2 tumors. Although the incidence was under 10% in both pT1a and pT1b1 tumors, it was relatively high in pT1b2 tumors compared with previous reports. Of the clinicopathological variables studied, only lymphatic invasion in pT1b2 tumors had a significant association with lymph node invasion. These results showed that the clinicopathological characteristics of pT1b1 tumors were more similar to those of pT1a tumors than those of pT1b2 tumors. We therefore combined pT1a and pT1b1 tumors in our analysis of relationships between histological types and nodal metastases. Mixed undifferentiated type tumors had a significantly higher incidence of nodal metastases than differentiated type tumors in both the pT1a-pT1b1 and the pT1b2 groups. Mixed histological type tumors have previously been reported to be a risk factor for nodal metastases [25], which is supported by our results showing that mixed undifferentiated type tumors are a risk factor for nodal metastases.

All four pT1a tumors and three of the pT1b1 tumors with nodal metastases in this study were signet-ring cell carcinomas with ulceration. The other pT1b1 tumor with nodal metastases was a differentiated type tumor without ulceration and without lymphatic or venous invasion. The 37 pT1b2 tumors with nodal metastases had varying histological findings. It seemed that depth of tumor invasion was the most important prognostic factor in these tumors.

We performed surgery for curative treatment of EGC in cases which were thought to have a possibility of nodal metastases. However, pathological diagnosis of the surgical specimens shows that many of these cases were overtreated by their surgery [26]. Accurate preoperative diagnosis of the presence or absence of lymph node metastases would simplify treatment decisions.

Preoperative and pathological tumor diagnoses may vary. The only part of the preoperative diagnosis which is almost definite is the histological type of the tumor. The accuracy of the preoperative diagnosis of depth of tumor invasion in mucosal tumors has been reported to be 80.2% [27]. Pathological findings after ESD show more detailed information and may indicate the need for additional treatment [28].

The accuracy of preoperative diagnosis of nodal metastases in EGC using computed tomography varies widely by methodology [29,30]. In this study, the accuracy of preoperative diagnosis was relatively low, and we did not know whether nodal metastases were present until we performed surgery with lymphadenectomy. We therefore selected treatment based mainly on the histological type of the tumor.

In general, we should currently perform surgery with adequate lymphadenectomy for EGC with an undifferentiated tumor type.

## Conclusions

Both endoscopic and surgical approaches are employed in the treatment of EGC. The aim of this study was to establish appropriate strategies for the treatment of EGC. We retrospectively examined the clinicopathological data of EGC patients who had undergone surgery. A total of 327 patients were eligible for the study, with a median follow-up period of 31 months. Nodal metastases were found in 4 of 161 patients with pT1a tumors; these were all signet-ring cell carcinomas with Type 0-IIc macroscopic appearance, and three of them did not have lymphatic or venous invasion. Nodal metastases were found in 4 of 43 patients with pT1b1 tumors and 37 of 123 patients with pT1b2 tumors. Lymph node metastases were significantly higher in mixed undifferentiated type group than differentiated type group for both groups, pT1a-pT1b1 (p = 0.0251) and pT1b2 (p = 0.0430) subgroups. The sensitivity of preoperative diagnosis of nodal metastases was 8.9% and the specificity was 96.1%. Nine patients with pT1b tumors had recurrence after surgery, with the initial sites of recurrence being liver, bone, peritoneum, distant nodes, and the surgical anastomosis. As the accuracy of preoperative diagnosis of nodal metastases was relatively low, we should at present perform surgery with adequate lymphadenectomy for undifferentiated type EGC.

## Abbreviations

DFS: disease-free survival; OS: overall survival; UICC: International Union Against Cancer

muc: mucinous adenocarcinoma; por: poorly differentiated adenocarcinoma; sig: signet-ring cell carcinoma; tub1: well differentiated adenocarcinoma; tub2: moderately differentiated adenocarcinoma

## Competing interests

The authors declare that they have no competing interests.

## Authors' contributions

HI* conceived and designed the study, collected clinical data, and performed the statistical analysis and interpretation of data. HI participated in the study design and performed interpretation of data. HI, MO, AY, TH, and KS collected clinical data. NE, RM, and NS participated in the study design and performed interpretation of data. CM and YW collected clinical data. NS participated in the study design and performed interpretation of data. SH delivered patients' pathologic data. SK participated in the study design and coordination. All authors read and approved the final manuscript.
